# Monoclonal antibodies neutralizing alpha-hemolysin, bicomponent leukocidins, and clumping factor A protected against *Staphylococcus aureus*-induced acute circulatory failure in a mechanically ventilated rabbit model of hyperdynamic septic shock

**DOI:** 10.3389/fimmu.2023.1260627

**Published:** 2023-09-15

**Authors:** Nhu T. Q. Nguyen, Thien N. M. Doan, Kei Sato, Christine Tkaczyk, Bret R. Sellman, Binh An Diep

**Affiliations:** ^1^ Division of HIV, Infectious Diseases, and Global Medicine, Department of Medicine, University of California, San Francisco, San Francisco, CA, United States; ^2^ Early Vaccines and Immune Therapies, AstraZeneca, Gaithersburg, MD, United States

**Keywords:** *Staphylococcus aureus*, mechanical ventilated rabbit model, hyperdynamic septic shock, neutralizing monoclonal antibodies, alpha-toxin, Panton-Valentine leukocidin, leukocidin, clumping factor A

## Abstract

**Background:**

Patients with septic shock caused by *Staphylococcus aureus* have mortality rates exceeding 50%, despite appropriate antibiotic therapy. Our objectives were to establish a rabbit model of *S. aureus* septic shock and to determine whether a novel immunotherapy can prevent or halt its natural disease progression.

**Methods:**

Anesthetized rabbits were ventilated with lung-protective low-tidal volume, instrumented for advanced hemodynamic monitoring, and characterized for longitudinal changes in acute myocardial dysfunction by echocardiography and sepsis-associated biomarkers after *S. aureus* intravenous challenge. To demonstrate the potential utility of this hyperdynamic septic shock model for preclinical drug development, rabbits were randomized for prophylaxis with anti-Hla/Luk/ClfA monoclonal antibody combination that neutralizes alpha-hemolysin (Hla), the bicomponent pore-forming leukocidins (Luk) including Panton-Valentine leukocidin, leukocidin ED, and gamma-hemolysin, and clumping factor A (ClfA), or an irrelevant isotype-matched control IgG (c-IgG), and then challenged with *S. aureus*.

**Results:**

Rabbits challenged with *S. aureus*, but not those with saline, developed a hyperdynamic state of septic shock characterized by elevated cardiac output (CO), increased stroke volume (SV) and reduced systemic vascular resistance (SVR), which was followed by a lethal hypodynamic state characterized by rapid decline in mean arterial pressure (MAP), increased central venous pressure, reduced CO, reduced SV, elevated SVR, and reduced left-ventricular ejection fraction, thereby reproducing the hallmark clinical features of human staphylococcal septic shock. In this model, rabbits pretreated with anti-Hla/Luk/ClfA mAb combination had 69% reduction in mortality when compared to those pretreated with c-IgG (*P*<0.001). USA300-induced acute circulatory failure—defined as >70% decreased in MAP from pre-infection baseline—occurred in only 20% (2/10) of rabbits pretreated with anti-Hla/Luk/ClfA mAb combination compared to 100% (9/9) of those pretreated with c-IgG. Prophylaxis with anti-Hla/Luk/ClfA mAb combination halted progression to lethal hypodynamic shock, as evidenced by significant protection against the development of hyperlactatemia, hypocapnia, hyperkalemia, leukopenia, neutropenia, monocytopenia, lymphopenia, as well as biomarkers associated with acute myocardial injury.

**Conclusion:**

These results demonstrate the potential utility of a mechanically ventilated rabbit model that reproduced hallmark clinical features of hyperdynamic septic shock and the translational potential of immunotherapy targeting *S. aureus* virulence factors for the prevention of staphylococcal septic shock.

## Introduction

Centers for Disease Control estimated 119,247 cases of *S. aureus* bacteremia (SAB) occurred in 2017 in United States, with 19,832 (16.6%) associated deaths (1). CDC reported an even higher mortality rate of 55.6% for patients with septic shock (2). Numerous bacterial virulence factors are implicated in animal models as contributing to the pathogenesis of *S. aureus* sepsis, such as surface proteins that facilitate tissue adherence and immune evasion, immunomodulatory proteins and coagulases that alter the inflammatory-coagulation interface, exotoxins that damage immune cells, endothelial cells, as well as different cells of the heart, lungs, liver and kidneys (3–7). Various clinical trials targeting some of these *S. aureus* virulence factors, such as its capsular polysaccharides, iron surface determinant B, manganese transport protein C, or clumping factor A (ClfA), have failed to demonstrate efficacy (8–11).

Results from clinical trials targeting pore-forming toxins by passive immunization with single monoclonal antibodies (mAb) are mixed. Although a cross-reactive mAb, ASN100, that neutralizes alpha-hemolysin (Hla) as well as two-component pore-forming toxins (Luk)—including Panton-Valentine leukocidin F component (LukF), leukocidin D, and gamma-hemolysin B component (HlgB)—protected against lethal challenge with a highly virulent community-associated methicillin-resistant *S. aureus* strain USA300 in mouse and rabbit pneumonia models (12), its phase 2 trial was terminated for futility after a planned interim analysis (13, 14). An anti-Hla mAb, MEDI4893/suvratoxumab, that protected against lethal challenge with USA300 in ferret and mouse pneumonia models but only partial protection in the rabbit pneumonia model showed a modest 31.9% (90% CI, -7.5% to 56.8%) relative risk reduction of pneumonia in a phase 2 clinical trial (15). It now seems imprudent to target only pore-forming toxins when staphylococcal pathogenesis is widely thought to be due to many virulence determinants (3–7).

The present study was undertaken to evaluate prophylactic efficacy of an anti-Hla/Luk/ClfA mAb combination in a newly developed mechanically ventilated rabbit model of USA300-induced septic shock. The anti-Hla/Luk/ClfA mAb combination was shown recently to confer protection in mouse models of surgical site infection (16), a diabetic mouse wound infection model (17), and a rabbit model of prosthetic joint infection (18). Mouse sepsis models are often used for preclinical testing but not used to evaluate efficacy of the anti-Hla/Luk/ClfA mAb combination because mouse polymorphonuclear leukocytes (PMNs) are known to be largely resistant to Panton-Valentine leukocidin and gamma-hemolysin and susceptible only to leukocidin ED (12). In contrast, rabbit and human PMNs were highly sensitive to all these cytotoxins (12, 19, 20), thereby making the rabbit a more suitable species for preclinical efficacy testing of the anti-Hla/Luk/ClfA mAb combination. Also, mouse sepsis models were not useful in predicting clinical efficacy of *S. aureus* conjugate capsular polysaccharides (21) and iron surface determinant B (IsdB) (22–24) vaccines that ultimately failed in phase 3 clinical trials (8, 25). The translational value of mouse sepsis models may be limited by the predominance of a hypodynamic circulation, which is typically not observed in septic shock patients (26–29). Because septic shock patients who were resuscitated adequately with fluids consistently demonstrated a hyperdynamic circulation with a high cardiac output (CO) and low systemic vascular resistance (SVR) (26–29), the recently published Minimum Quality Threshold in Preclinical Sepsis Studies *guideline* recommended that preclinical sepsis models should administer fluid resuscitation in order to separate sepsis-related events from pathological events resulting solely from progressive circulatory deterioration due to protracted hypovolemia (30). Fluid resuscitation was implemented successfully in non-human primate, canine and mouse Gram-negative sepsis models, which was critical for the development of both hyperdynamic state and myocardial depression seen in human sepsis (31–34). Unfortunately, fluid resuscitation was not implemented in commonly used mouse *S. aureus* sepsis models (21–24), likely because assessment of fluid responsiveness and its impact on sepsis-induced changes in cardiovascular performance require advanced hemodynamic monitoring and/or echocardiography, methods which are not available in most research laboratories.

To develop rabbit models that better predict the human clinical response and support drug regulatory approval, we used funding from the Food and Drug Administration to establish an experimental ICU to perform pathophysiology and preclinical efficacy studies to closely mimic clinical practice (35, 36). We describe here a new preclinical model of hyperdynamic septic shock induced by *S. aureus* in which rabbits are mechanically ventilated, instrumented for advanced hemodynamic monitoring (MAP, central venous pressure, cardiac output, stroke volume, systemic vascular resistance), evaluated for acute myocardial dysfunction by echocardiography, and assessed for changes in sepsis-associated blood biomarkers using real-time point-of-care instruments. In a natural history study, rabbits challenged intravenously with USA300 developed hallmark clinical features of a hyperdynamic state of septic shock that was followed by a short-lived and lethal hypodynamic state in which animals developed global left-ventricular dysfunction and acute circulatory failure. In a prophylactic efficacy study, rabbits pretreated with the anti-Hla/Luk/ClfA mAb combination, but not those pretreated with irrelevant isotype-matched control mAb (c-IgG), were protected against the lethal course of USA300-induced septic shock.

## Methods

### *S. aureus* preparation

A minimal-passaged SF8300 clinical strain representative of the epidemic clone USA300-0114 was used to induce lethal septic shock in the mechanically ventilated rabbit model. SF8300 was grown in 10 mL of tryptic soy broth (TSB) media (Sigma-Aldrich) overnight, then diluted 1:50 into 50 mL of TSB media in a 250 mL Erlenmeyer flask. Bacteria were grown at 37°C with shaking at 120 RPM and harvested at an OD_600 nm_ of 0.4. Cells were washed and resuspended in sterile phosphate-buffered saline (PBS) to a concentration of 1 × 10^10^ colony-forming units (CFU)/mL, aliquoted into individual cryovials, and immediately stored at -80°C (19). Frozen stocks were titered in triplicate on three separate occasions before use in any experiment. Based on this value, the inoculum containing 9.4 x 10^8^ CFUs (in 2.2 mL saline solution) was prepared and again tittered in triplicate to document the numbers of bacterial CFUs used for intravenous challenge of rabbits.

### Monoclonal antibodies

Anti-Hla mAb (37), anti-Luk mAb that cross-neutralizes LukF-PV (PVL), LukD (leukocidin ED), HlgB (γ-hemolysin) (16), and anti-ClfA mAb (38) were generated as described previously. An irrelevant isotype-matched control IgG (c-IgG) directed against the gp120 protein of HIV was used as control (37).

### Mechanically ventilated rabbit model of USA300-induced septic shock

The rabbit model was reviewed and approved by the University of California San Francisco Institutional Animal Care and Use Committee. Experiments were conducted in a facility certified by the Association for Assessment and Accreditation of Laboratory Animal Care International. New Zealand White rabbits (Western Oregon Rabbit Co.), 3.4 - 4.0 kg, 16 - 20 weeks of age, both male or female rabbits, were sedated by administration of buprenorphine (0.01-0.05 mg/kg) subcutaneously, and then, after 30 minutes, injected intramuscularly with a solution containing 36 mg/kg ketamine and 5.2 mg/kg xylazine. Ophthalmic ointment was placed on each eye. Once the desired depth of anesthesia was achieved as assessed by lack of pedal reflex, a 3.0 mm cuffed endotracheal tube was introduced orally into the trachea. The endotracheal tube was connected to a neonatal ventilatory circuit (Fisher & Paykel), connected to a heated humidifier (Fisher & Paykel) and then a ventilator (ADS2000, Engler) with the following settings: peak inspiratory pressure (PIP) of 12-15 cm H_2_O, positive end-expiration pressure (PEEP) of 5 cmH_2_O, respiratory rate of 30 breaths/min, flow rate of 4 L/min, fraction of inspired oxygen (FiO_2_) of 0.35, and 2.0-2.5% isoflurane to maintain general anesthesia. The ventilator settings remain constant for the duration of the study, except when FiO_2_ is increased to 1.0 when performing suctioning for removal of mucus that built up at the distal end of the endotracheal tube. The respiratory rate may be adjusted to maintain PaO_2_ between 140 and 200 mmHg and PaCO_2_ between 35 and 45 mmHg. The left or right marginal ear vein was catheterized with a 22G x 1” IV catheter for continuous infusion of Normosol-R with 5% dextrose (4 mL/kg/h) using an infusion pump (Hospira) for fluid maintenance.

The right carotid artery was catheterized with either 4 French pediatric PiCCO catheter (Getinge) for the natural history study or 18G arterial catheter (Argon Medical Devices) for the efficacy study and positioned approximate 2-3 cm from the common aortic arch, whereas the right internal jugular vein was catheterized with a 4 French dual-lumen pediatric central venous catheter (Medline) and positioned 1-2 cm from the right atrium using a common cutdown approach as detailed here. The surgical site was shaved, scrubbed, and prepped aseptically. The surgical site was draped out with sterile towels. The rabbit was placed in a supine position and depth of anesthesia was assessed by pinching between the toes. If rabbit was responsive, isoflurane was increased to effect. A 2-3 cm incision was made to access the right carotid artery/internal jugular vein using aseptic technique. Blunt and sharp dissection was used to identify and isolate the carotid artery from the Vagus nerve as well as the internal jugular vein. A distal 3-0 silk ligature was placed around the carotid artery/jugular vein to prevent back bleeding. A loose proximal ligature was placed around the carotid artery/jugular vein. A small (approximately 1 mm) cut was made at an angle across the vessel and the catheter was passed through the cut proximally and advanced about 5 cm so that the catheter tip was positioned approximately 2-3 cm from the common aortic arch or 1-2 cm from the right atrium. The proximal ligature was tied to secure the catheter in place. The vessels were repositioned and the wound was closed in at least 2 layers. The catheters were connected to pressure transducers with integrated flush device (Getinge) and the patient monitor (MP90, Philips Healthcare) for continuous blood pressure monitoring. To maintain patency, the catheters were continuously flushed with 3 ml/h normal saline containing 1U/ml heparin, and manually flushed every 1 h with three quick flushes of normal saline containing 1U/ml heparin.

Once the catheters were in place, the transpulmonary thermodilution method was used to measure cardiac output every 2 h by injecting a bolus of 2-mL cold normal saline through the central venous catheter. A thermistor located at the tip of the PiCCO catheter recorded the very brief drop in the blood temperature for calculation of a thermodilution curve and cardiac output (CO) on the patient monitor (MP90, Philips Healthcare). For each timepoint, CO was measured two times about 3-4 min apart, and then averaged. If the two CO measurements differed >10% from one another, a third CO measurement was performed, and the three measurements averaged. At 6 h after surgery, rabbits were challenged intravenously via the IV catheter placed in the marginal ear vein with a 2.2 ml saline solution containing 9.4 x 10^8^ CFUs of USA300.

### Echocardiography

Two-dimensional echocardiography was used to assess global left ventricular hypokinesia in the natural history study rabbits. Images were obtained with a cardiac ultrasound machine (iE33, Philips Healthcare) equipped with S12-4 sector array pediatric transducer. We measured the left ventricular and auricular dimensions as well as left ventricular ejection fraction (LVEF) using the parasternal long axis view of the heart via Teicholz method. 

### Serial blood sampling and analysis

Arterial blood samples were collected at pre-infection baseline (within 1 h of intravenous challenge with USA300), and then every 2 h for the first 24 h post infection (hpi) and every 4 h until the end of the study. Blood gas analysis was determined using RapidPoint 500 (Siemens), including partial pressure of oxygen (PaO_2_) and of carbon dioxide (PaCO_2_), bicarbonate [HCO_3_^-^]), base excess, sodium (Na^+^), potassium (K^+^), chloride (Cl^-^), calcium (Ca^++^), glucose, and lactate. The Element HT5 Veterinary Hematology Analyzer^©^ (Heska, Loveland, CO) was used to determine complete blood count with five-part differential. VetScan VS2^©^ (Abaxis, Union City, CA) was used for analysis of alanine aminotransferase (ALT), aspartate aminotransferase (AST), creatinine and blood urea nitrogen.

### Microbiology and tissue sample processing

Heart, lung, liver, kidney, and spleen were weighed and processed for bacterial count. For each organ, 3 to 4 pieces (0.05-0.10 grams each) were removed from different parts of the organ, totaling 0.1-0.2 grams, and then homogenized for 30-90 seconds using a Tissue-Tearor (BioSpec, Bartlesville, OK). Each homogenate (100 µL) was then serially diluted in 900 µL of normal saline and 100 µL of each dilution plated onto 5% blood sheep agar and incubated for 16-24 h at 37°C for CFU count.

### Statistical analysis

Our hypothesis is that survival of rabbits pretreated with c-IgG is shorter than survival of those pretreated with anti-Hla/Luk/ClfA mAb combination in a rabbit model of USA300-induced septic shock. We calculated that a sample size of 9 animals per experimental group would provide a power of 80% to detect a hazard ratio of 0.25, with a two-sided type I error 0.05 by means of a log-rank test (Schoenfeld method) using STATA version 17.0 (StataCorp). The sample size was increased to 10 animals per experimental group to account for potential exclusion of animals in this model for technical problems related to intubation, surgery for placement of catheter in the carotid artery, or occlusion of endobronchial tube from mucus buildup during mechanical ventilation (35). Survival curves were generated using the Kaplan-Meier method, and significance was assessed by means of the log rank (Mantel-Cox) test. Nonparametric statistics, median [25^th^ percentile – 75^th^ percentile], were computed for all variables. Bacterial count and lung weight/body weight (LW/BW) ratio were compared using a nonparametric two-sided Mann-Whitney U test. Two-sided Fisher’s exact test was used to determine whether rabbits pretreated with c-IgG or anti-Hla/Luk/ClfA mAb combination differ in the proportions of categories for the various blood tests (e.g., hypocapnia). Nonparametric one-way analysis of variance (ANOVA) with Kruskal-Wallis test followed by Dunn’s multiple comparison test were used to evaluate effect of pretreatment with c-IgG or anti-Hla/Luk/ClfA mAb combination on blood tests conducted at pre-infection baseline or terminal endpoint.

## Results

### Natural history study revealing a hyperdynamic state of USA300-induced septic shock in mechanically ventilated rabbits

**Figure 1:**
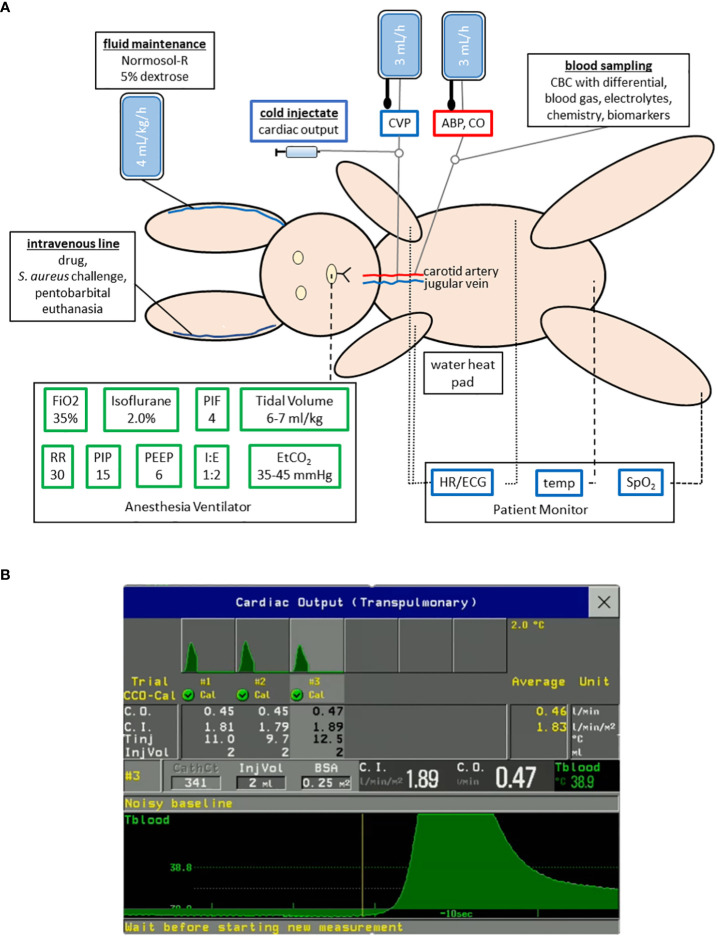
Experimental setup for a mechanically ventilated rabbit model of hyperdynamic septic shock with advanced hemodynamic monitoring. **(A)** Rabbits were anesthetized with isoflurane and mechanically ventilated with lung-protective low-tidal volume parameters as shown. A thermodilution catheter placed in the right carotid artery and central venous catheter (CVC) in the right internal jugular vein for continuous measurement of arterial blood pressure (ABP), central venous pressure (CVP), and heart rate (HR), as well as intermittent measurement of cardiac output. **(B)** Changes in blood temperature (Tblood) were measured by transpulmonary thermodilution of cold saline of known volume (InjVol) and temperature (Tinj) that was injected through the CVC and passed through the right heart, the lungs, and the left heart and then detected by the PiCCO catheter placed in the carotid artery. This procedure was repeated 2-3 times within 10 minutes to ensure that an accurate average of the cardiac output (C.O.), which is inversely related to the area under the thermodilution curve, was computed on the cardiac monitor (Philips Intellivue MP90 with PiCCO module).

We performed a natural history study to characterize the pathogenesis of USA300-induced septic shock in anesthetized rabbits that were intubated for mechanical ventilation with a lung-protective low-tidal volume (**Figure 1A**), as previously described (35). Anesthetized rabbits were instrumented with the PiCCO catheter and central venous catheter for advanced hemodynamic monitoring, including intermittent measurement of cardiac output by transpulmonary thermodilution method (**Figures 1A, B** **)**. Rabbits were infused with a total of 60-65 mL/kg of a balanced crystalloid solution over the course of 6 h preceding *S. aureus* challenge for (*i*) fluid maintenance at 4 mL/kg/h, (*ii*) continuous flushing the arterial and central venous catheters at 3 mL/h each (**Figure 1A**), and (*iii*) fluid resuscitation at 30 mL/kg to counteract the hypotension associated with overstimulation of Vagus nerve during carotid artery catheterization (35), thereby providing adequate volume repletion that may be crucial for the development of a hyperdynamic state of septic shock after *S. aureus* challenge.

**Figure 2:**
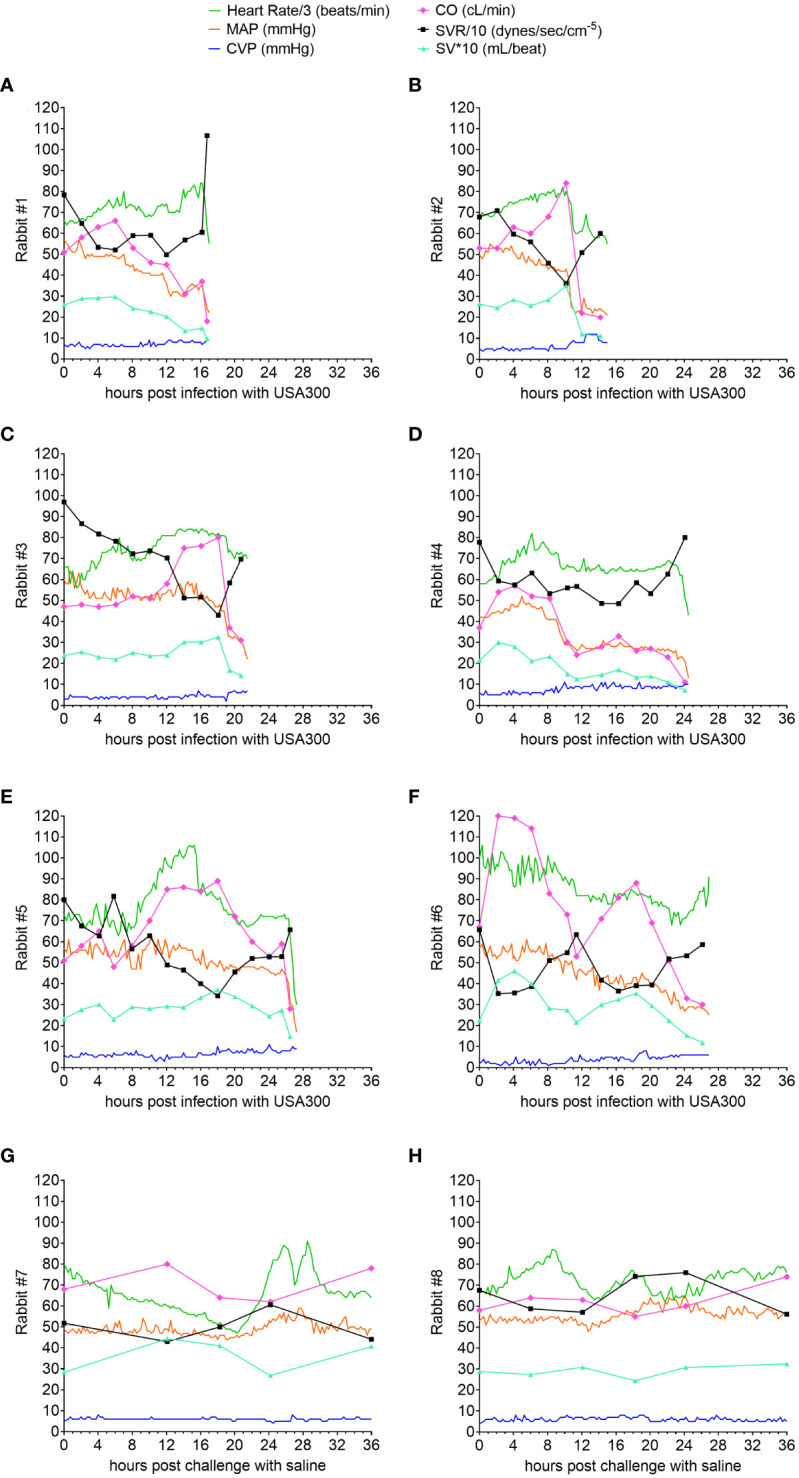
Natural history study showing hyperdynamic and hypodynamic states of USA300-induced septic shock in mechanically ventilated rabbits. Advanced hemodynamic monitoring was performed for rabbits challenged with USA300 **(A–F)** or saline **(G, H)**. Heart rate (HR), mean arterial pressure (MAP) and central venous pressure (CVP) were recorded every 15 minutes, whereas cardiac output (CO) was determined intermittently at the time points indicated in each graph. Systemic vascular resistance (SVR) and stroke volume (SV) were computed based on HR, MAP, CVP and CO. Numerical values for HR were divided by 3 and SVR by 10, and SV multiplied by 10 so that they can be displayed with the other parameters using the same scale on the y-axis.

Mortality rate was 100% (6/6) for USA300-challenged rabbits, with acute circulatory failure and death occurring between 15 and 27 h post infection (**Figures 2A–F**), whereas the two saline-challenged rabbits survived to the end of the study period at 36 h post challenge (**Figures 2G, H** **)**. The hyperdynamic state of USA300-induced septic shock, which was characterized by elevated cardiac output, increased stroke volume, and decreased systemic vascular resistance, occurred early at 2-6 h post infection in five of the rabbits (**Figures 2A, B, D–F** **)** or later at 8-18 h post infection in one rabbit (**Figure 2C**), thereby reproducing the clinical hyperdynamic state with increased heart rate, elevated cardiac output, increased stroke volume, and decreased systemic vascular resistance in adequately volume-resuscitated patients with septic shock (26–29). Then the ensuing hypodynamic state, which was characterized by decreased heart rate, decreased MAP, increased central venous pressure, decreased cardiac output, decreased stroke volume, and elevated systemic vascular resistance, occurred in the 4-6 h period before the rabbits succumbed to USA300-induced septic shock (**Figures 2A–F**), mimicking the terminal phase in non-survivor patients with hypodynamic shock and death (39). MAP, central venous pressure, cardiac output, stroke volume and systemic vascular resistance remained within normal limits for the two saline-challenged rabbits, consistent with our previous findings that our lung-protective low-tidal volume mechanical ventilation protocol and instrumentation did not negatively impact rabbits without infectious challenge (35).

### Acute myocardial dysfunction in rabbits during hypodynamic shock

**Figure 3:**
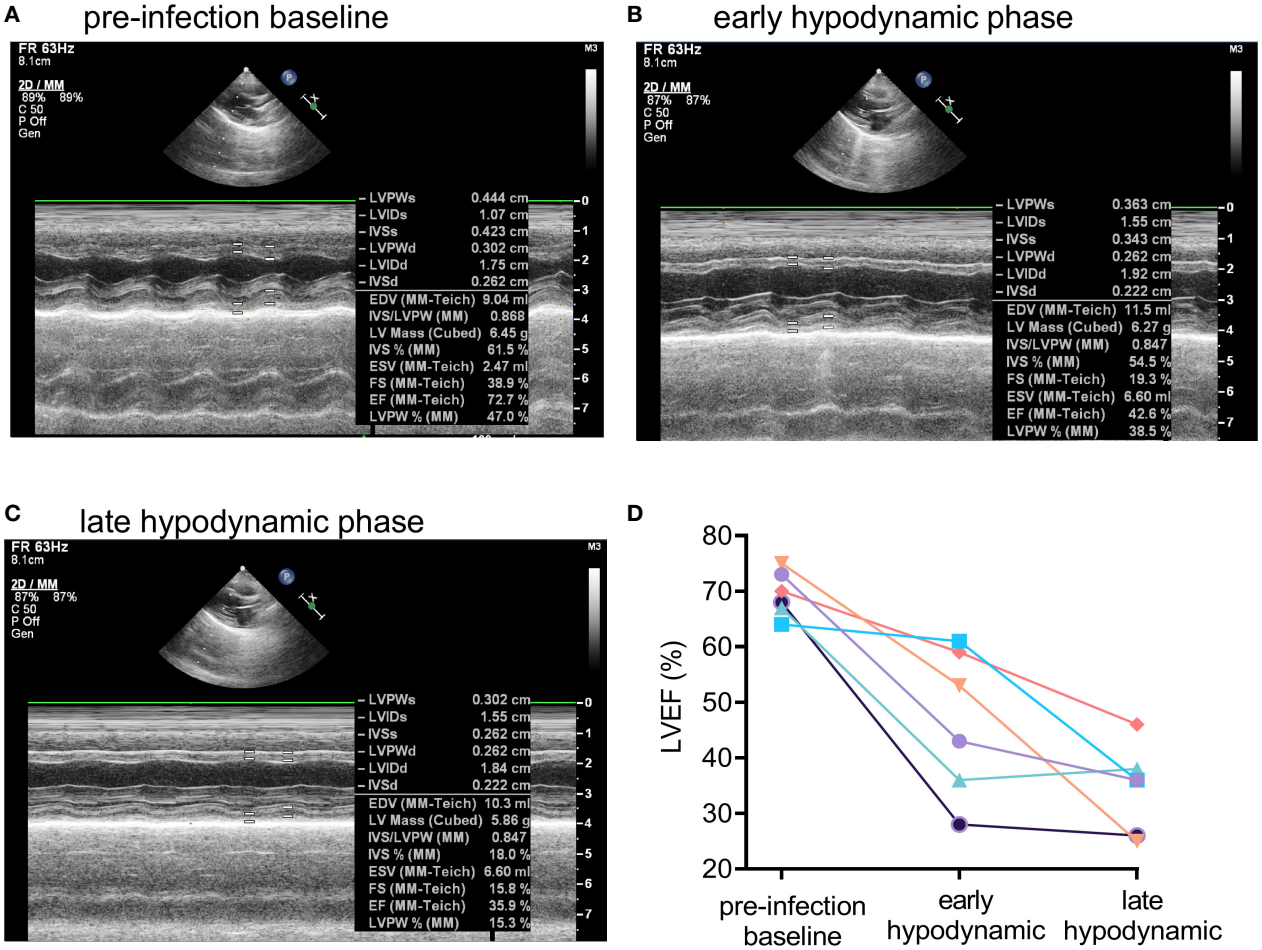
Development of global left ventricular dysfunction in early and late hypodynamic states of USA300-induced septic shock. Representative M-mode echocardiographic tracing showing systolic and diastolic left ventricular dimensions in a USA300 WT-infected for Rabbit #1 at **(A)** pre-infection baseline, **(B)** early hypodynamic state when MAP decreased by about 20% from pre-infection baseline, and **(C)** late hypodynamic state in the when MAP was decreased >30% from pre-infection baseline. **(D)** Left ventricular ejection fraction (LVEF) for six rabbits challenged with USA300 declined significantly from pre-infection baseline to the early and then late hypodynamic states (linear test for trend, *P* < 0.001). For two control rabbits challenged with saline, LVEF values were unchanged over time: 67%, 69%, 64% for Rabbit #7, and 64%, 62%, and 62% for Rabbit #8 at pre-infection baseline, 5 h post saline challenge, and 36 h post saline challenge, respectively.

Transthoracic echocardiography was used in the rabbit natural history study to characterize further potential septic shock-induced acute myocardial dysfunction, including global left ventricular hypokinesia, which occurred in 60% of mechanically ventilated patients with septic shock (40). Representative M-mode echocardiographic tracings for Rabbit #1 (see **Figure 2A** for corresponding changes in hemodynamic parameters) obtained at pre-infection baseline (**Figure 3A**), at the early hypodynamic state when MAP decreased 20% from pre-infection baseline value (**Figure 3B**), and at the late hypodynamic state when MAP decreased >30% from pre-infection baseline value (**Figure 3C**) illustrated the development of progressive global left ventricular hypokinesia. For all six rabbits with USA300-induced septic shock, left ventricular ejection fraction (LVEF) was 70% (range from 64% to 75%) at pre-infection baseline, and decreased progressively to 47% (28% to 61%) during the early hypodynamic state, and then to 35% (25% - ;46%) during late hypodynamic state (test for linear trend *P*<0.001; **Figure 3D**).

### Prophylactic efficacy of anti-Hla/Luk/ClfA mAb in a rabbit model of USA300-induced septic shock

**Figure 4:**
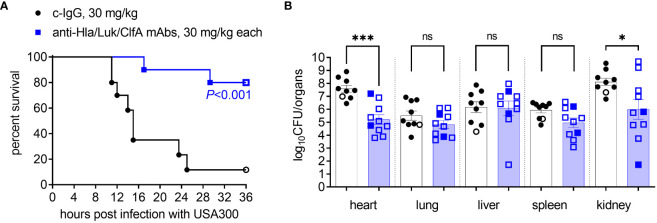
Anti-Hla/Luk/ClfA mAb combination improved survival in rabbit model of USA300-induced hyperdynamic septic shock. **(A)** Kaplan-Meier survival curves for rabbits pretreated with indicated mAbs and then challenged intravenously with 9.4 x 10^8^ CFUs of USA300. Two-sided Mantel-Cox log-rank test was used to compare survival rates with *P*<0.05 considered statistically significant. **(B)** Bacterial counts of vital organs for rabbits determined at the terminal endpoint when rabbits succumbed to infection (filled symbol) or at the end of the monitoring period 36 h post infection (open symbol). Nonparametric Mann-Whitney U test was used to evaluate differences in bacterial counts in vital organs for animals pretreated with c-IgG vs. anti-Hla/Luk/ClfA antibody. *P*-values ≥0.05 are marked as ns for not significant, * if <0.05, and *** if <0.001.

Rabbits were randomized for intravenous injection with either 30 mg/kg each of the anti-Hla/Luk/ClfA mAb combination or irrelevant isotype-matched c-IgG. After 16-20 h, rabbits were challenged intravenously with the community-associated MRSA USA300, which is known to produce the virulence determinants targeted by our mAb combination: α-hemolysin is neutralized by anti-Hla mAb; F components of the bicomponent toxin LukF-PV (PVL), LukD (leukocidin ED), HlgB (γ-hemolysin) are cross-neutralized by anti-Luk mAb (41); and clumping factor A is neutralized by anti-ClfA mAb. Overall survival rates were 11% (1/9) for rabbits pretreated with c-IgG compared to 80% (8/10) for those pretreated with anti-Hla/Luk/ClfA mAb combination (*P*<0.001 by log-rank test; **Figure 4A**).

*S. aureus* quantified in heart and kidney samples showed significant log_10_CFU reduction (*P*<0.001 and *P*=0.043 by nonparametric Mann-Whitney test), whereas those in lung, spleen, kidney, and liver samples showed non-significant reduction in rabbits pretreated with anti-Hla/Luk/ClfA mAb combination compared to those pretreated with c-IgG (**Figure 4B**).

### Anti-Hla/Luk/ClfA mAb combination protected against acute circulatory failure

**Figure 5:**
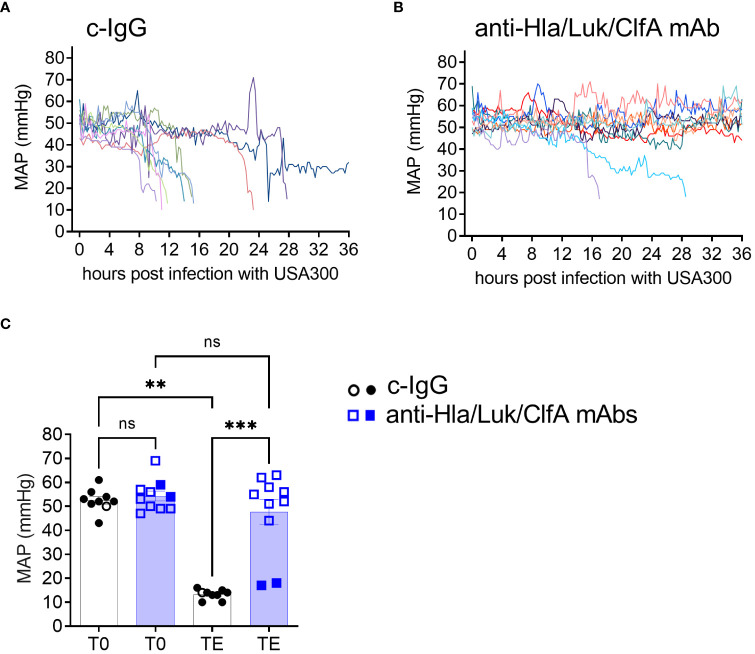
Anti-Hla/Luk/ClfA mAb combination, but not c-IgG, protected against acute circulatory failure. Mean arterial pressure (MAP) were recorded every 15 minutes in rabbits pretreated with **(A)** c-IgG or **(B)** anti-Hla/Luk/ClfA mAb combination. **(C)** MAP at pre-infection baseline (T0) or terminal endpoint (TE) for the two experimental groups were compared by nonparametric one-way analysis of variance (ANOVA) with Kruskal-Wallis test followed by Dunn’s multiple comparison tests. Data points from rabbits that succumbed to infection are represented by filled symbols, and those euthanized at the end of the monitoring period 36 h post infection by open symbols. *P*-values ≥0.05 are marked as ns for not significant, ** if <0.01, and *** if <0.001.

Although cardiac output and central venous pressure were measured in the natural history study, these advanced hemodynamic parameters were not measured for the efficacy study of the anti-Hla/Luk/ClfA mAb combination because they are labor intensive, and the more expensive thermodilution and central venous catheters that we used are more prone to thrombotic occlusion due to their narrower tip design, thereby requiring removal and exchange with new catheters in 20-30% of rabbits. Nonetheless, for the efficacy study, we catheterized the right carotid artery for continuous blood pressure monitoring with a similarly sized arterial catheter in which the narrow tip was cut off with a sharp surgical blade, creating a 90° blunt tip which helped reduce the frequency of thrombotic catheter occlusion to <5% of rabbits. Mean arterial pressure (MAP) was recorded every 15 min, providing a high-resolution view of the individual rabbit’s hemodynamics (**Figures 5A, B** **)**. MAP declined sharply in all (9/9) c-IgG-pretreated rabbits in the 2-4 h preceding death (**Figure 5A**), corresponding to the hypodynamic state of septic shock described above (**Figures 2**, **3**), whereas MAP remained within normal limits for 80% (8/10) of anti-Hla/Luk/ClfA mAb combination-pretreated rabbits that survived to the end of the study at 36 h post infection and declined sharply in only the remaining 20% (2/10) of rabbits that died (**Figure 5B**). The one survivor pretreated with c-IgG developed severe hypotension at 25 h post infection when MAP decreased to 14 mmHg, but then increased rapidly and remained from 25 to 32 mmHg until the animal was euthanized at 36 h post infection, the end of the study period (**Figure 5A**). USA300-induced acute circulatory failure, defined as >70% decreased in MAP from pre-infection baseline, occurred in 20% (2/10) of rabbits pretreated with anti-Hla/Luk/ClfA mAb combination compared to 100% (9/9) rabbits pretreated with c-IgG compared to (two-sided Fisher’s exact test, *P* < 0.001; see also **Figure 5C**).

### Hypoxemic respiratory failure not a consistent feature of this rabbit model of USA300-induced septic shock

**Figure 6:**
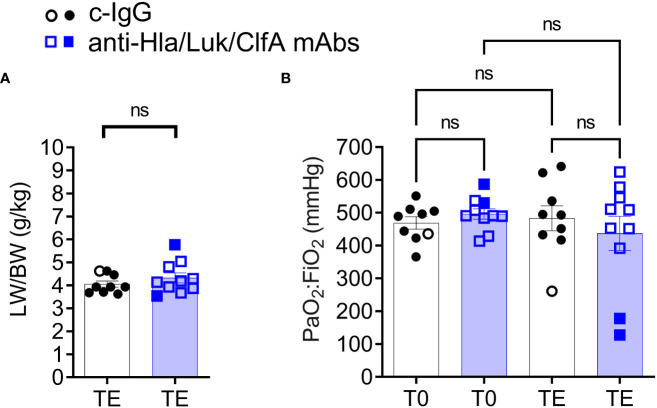
Intravenous challenge with USA300 did not cause hypoxemic respiratory failure. **(A)** Lung weight to body weight (LW/BW) ratio determined at pre-infection baseline (T0) or the terminal endpoint (TE) when rabbits succumbed to infection (filled symbol) or at the end of the monitoring period 36 h post infection (open symbol). Nonparametric Mann-Whitney U test was used to evaluate differences in LW/BW pretreated with c-IgG compared to anti-Hla/Luk/ClfA antibody. **(B)** PaO_2_:FiO_2_ determined at pre-infection baseline (T0) or TE and compared by nonparametric one-way analysis of variance (ANOVA) with Kruskal-Wallis test followed by Dunn’s multiple comparison tests. *P*-values ≥0.05 are marked as ns for not significant.

Despite USA300 infecting the lungs of all rabbits (**Figure 4B**), the lung weight to body weight (LW/BW) ratio in g/kg, a quantitative measure of acute lung injury, was 4.2 [3.8 – 4.9] for rabbits pretreated with anti-Hla/Luk/ClfA mAb combination and 3.9 [3.7 - 4.5] for those pretreated with c-IgG (Mann Whitney test, *P*=0.50; **Figure 6A**), which are similar to the LW/BW ratio of 3.9 for uninfected control rabbits that were managed using the same aforementioned lung-protective ventilatory strategies (35). Accordingly, hypoxemic respiratory failure was not a consistent feature of the rabbit model because the ratio of partial pressure arterial oxygen and fraction of inspired oxygen (PaO_2_/FiO_2_) decreased to 100 mmHg - 300 mmHg, thresholds for mild to moderate in ARDS severity according to the Berlin definition (42), in only 20% (2/10) and 11% (1/9) of rabbits pretreated with anti-Hla/Luk/ClfA mAb combination or c-IgG, respectively (two-sided Fisher’s exact test, *P* = 1.00; **Figure 6B**).

### Temporal changes in peripheral white blood cell populations and survival in rabbits pretreated with anti-Hla/Luk/ClfA mAb combination

**Figure 7:**
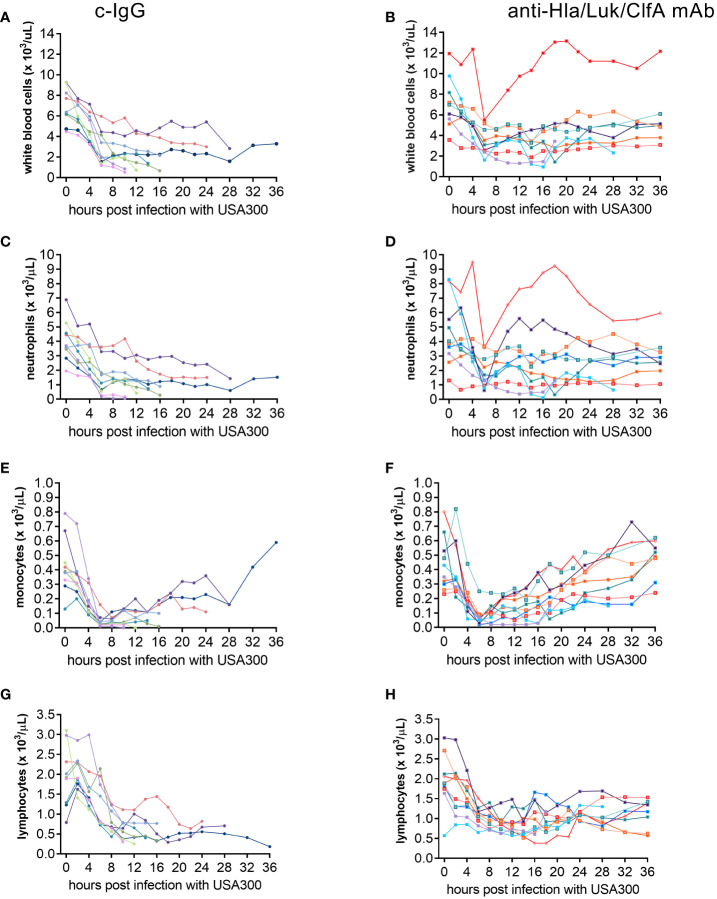
Temporal changes in circulating cell populations. Serial blood samples were collected from carotid artery and analyzed on a veterinary hematology analyzer for complete blood count with differential for rabbits pretreated with c-IgG **(A, C, E, G)** or anti-Hla/Luk/ClfA mAb combination **(B, D, F, H)**.

To determine whether temporal changes in circulating white blood cell populations are associated with survival of rabbits pretreated with anti-Hla/Luk/ClfA mAb combination or c-IgG, serial blood samples were collected from each rabbit at pre-infection baseline, 2, 4, 6, 8, 10, 12, 14, 16, 18, 20, 22, 24, 28, 32, 36 h post infection or within 1 h of the time when rabbit succumbed to infection for complete blood count with differential. Peripheral white blood cells, including neutrophils and monocytes, declined very rapidly within the first 6 h post infection for all rabbits, but then increased among the 80% (8/10) and 11% (1/9) of rabbits pretreated with anti-Hla/Luk/ClfA mAb combination or c-IgG, respectively, that survived to the end of the study at 36 h post infection (two-sided Fisher’s exact test, *P* = 0.006; **Figures 7A–F**). Among the 8 non-survivors pretreated with c-IgG, those with the steepest decline in white blood cells, neutrophils and monocytes also developed earliest acute circulatory failure and death (**Figures 7A, C, E** **)**. Lymphocyte counts also declined, but less rapidly compared to the decline of neutrophils and monocytes, in all rabbits from both experimental groups (**Figures 7G, H**). Compared to rabbits pretreated with c-IgG, those pretreated with anti-Hla/Luk/ClfA mAb combination had significantly greater numbers of white blood cells (4.9 [3.7 - 6.4] vs. 1.4 [0.7 - 2.9] * 10^3^/µL, *P*<0.001), neutrophils (2.5 [1.7 - 3.4] vs. 0.7 [0.2 - 1.5] * 10^3^/µL, *P*<0.001), monocytes (0.49 [0.29 - 0.56] vs. 0.05 [0.01 - 0.14] * 10^3^/µL, *P*=0.002), and lymphocytes (1.2 [0.9 - 1.4] vs. 0.4 [0.3 - 0.8] * 10^3^/µL, *P*=0.002) at the terminal endpoint (**Figures 7A–H**).

### Differences in biomarkers in rabbits pretreated with anti-Hla/Luk/ClfA mAb combination or c-IgG

**Figure 8:**
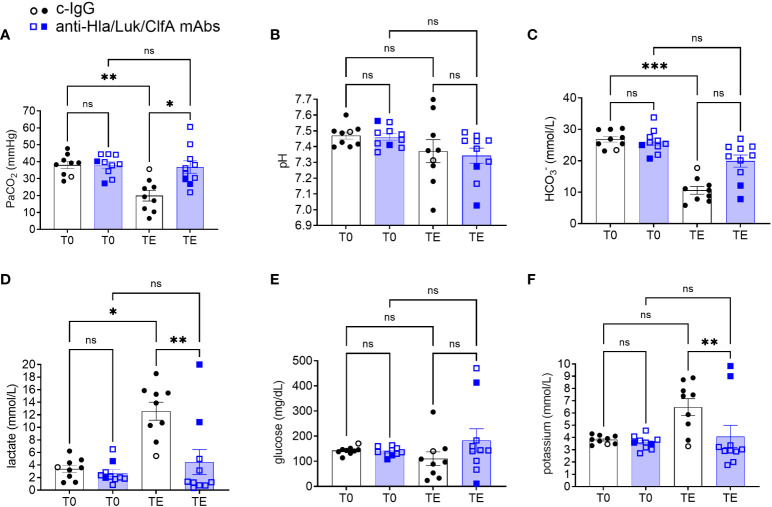
Arterial blood gas analysis in rabbits pretreated with anti-Hla/Luk/ClfA mAb combination or c-IgG. **(A)** Partial pressure of arterial carbon dioxide (PaCO_2_), **(B)** pH, **(C)** HCO_3_^-^, **(D)** lactate, **(E)** glucose, and **(F)** potassium determined at pre-infection baseline (T0) or the terminal endpoint (TE) within 1 h of when rabbits succumbed to infection or at 36 h post-infection at the end of the study period for rabbits pretreated with anti-Hla/Luk/ClfA mAb combination or c-IgG. Multiplicity adjusted *P*-values were calculated by nonparametric one-way analysis of variance (ANOVA) with Kruskal-Wallis test followed by Dunn’s multiple comparison test for the four comparisons indicated in each panel. *P*-values >0.05 are marked with ns for not significant, * for <0.05, ** for <0.01, and *** for <0.001.

Lactate at the terminal endpoint was 1.3 [0.8 - 6.4] mmol/L for rabbits pretreated with anti-Hla/Luk/ClfA mAb combination compared to 13.9 [9.0 - 15.7] mmol/L for those pretreated with c-IgG (multiplicity-adjusted *P*<0.01; **Figure 8D**). Lactate increased in all (10/10) non-survivors but remained similar to pre-infection baseline for 9/9 survivors from both experimental groups (**Figure 8D**), consistent with hyperlactatemia being one of the strongest prognostic biomarker of survival in patients with septic shock (43, 44).

Despite no differences at pre-infection baseline, PaCO_2_ at the terminal endpoint was 35 [29 - 44] mmHg for rabbits pretreated with anti-Hla/Luk/ClfA mAb combination compared to 19 [11 - 27] mmHg for those pretreated with c-IgG (multiplicity-adjusted *P*<0.05; **Figure 8A**). Severe hypocapnia, defined as PaCO_2_ < 25 mmHg, occurred in 10% (1/10) of rabbits pretreated with anti-Hla/Luk/ClfA mAb combination compared to 78% (7/9) of those pretreated with c-IgG (two-sided Fisher’s exact test, *P* = 0.005). This is consistent with findings in patients with septic shock in which severe hypocapnia was associated with death (45, 46).

pH showed broad changes, ranging from marked acidosis to alkalosis, in rabbits from both experimental groups, and did not correlate with survival or death (**Figure 8B**). In contrast, bicarbonate decreased to the lowest level in all (10/10) non-survivors compared 0/9 survivors, although differences between the two experimental groups at the terminal endpoint were not statistically significant (**Figure 8C**).

Glucose also displayed a broad range at the terminal endpoint for rabbits in both experimental groups, with hypoglycemia (<100 mg/dL) occurring in 37% (7/19) and hyperglycemia (>200 mg/dL) in 16% (3/19) of rabbits (**Figure 8E**). Potassium levels at the terminal endpoint was 3.0 [2.7 - 4.8] mmol/L for rabbits pretreated with anti-Hla/Luk/ClfA mAb combination compared to 7.4 [4.5 - 8.3] mmol/L for those pretreated with c-IgG (multiplicity-adjusted *P*<0.01; **Figure 8F**). Extreme hyperkalemia (>6.5 mEq/L) occurred in 70% (7/10) of rabbits from both experimental groups that succumbed to USA300-induced septic shock and none (0/9) of the survivors (**Figure 8F**).

### Changes in biomarkers associated with multiple organ dysfunction

**Figure 9:**
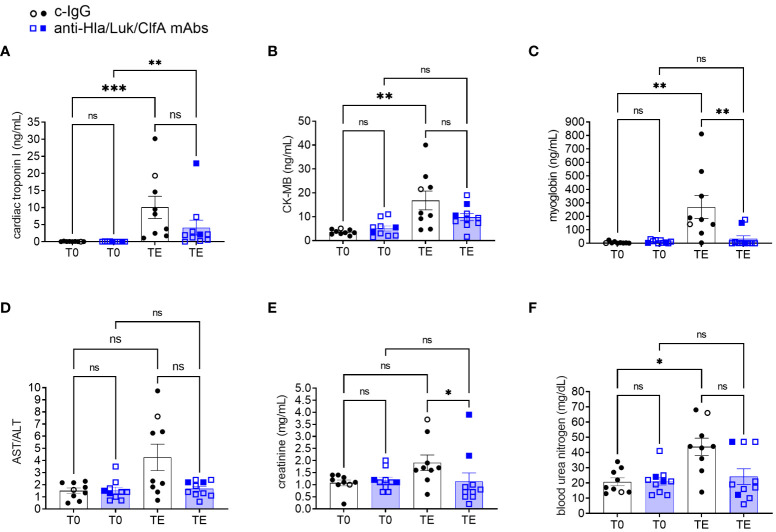
Biomarkers of multiple organ dysfunction in rabbits pretreated with anti-Hla/Luk/ClfA mAb combination or c-IgG. **(A)** Cardiac troponin I, **(B)** creatine kinase-MB (CK-MB), **(C)** myoglobin, **(D)** aspartate aminotransferase/alanine aminotransferase (AST/ALT), **(E)** creatinine, and **(F)** blood urea nitrogen determined at pre-infection baseline (T0) or the terminal endpoint (TE) within 1 h of when rabbits succumbed to infection or at 36 h post-infection at the end of the study period for rabbits pretreated with anti-Hla/F/ClfA mAb combination or c-IgG. Multiplicity adjusted *P*-values were calculated by nonparametric one-way analysis of variance (ANOVA) with Kruskal-Wallis test followed by Dunn’s multiple comparison test for the four comparisons indicated in each panel. *P*-values >0.05 are marked with ns for not significant, * for <0.05, ** for <0.01, and *** for <0.001.

Acute myocardial injury biomarkers, including cardiac troponin I, CK-MB, and myoglobin, increased significantly from pre-infection baseline to the terminal endpoint for rabbits pretreated with c-IgG (**Figures 9A–C**). In contrast, cardiac troponin I, but not CK-MB or myoglobin, increased significantly from pre-infection baseline to the terminal endpoint for rabbits pretreated with anti-Hla/Luk/ClfA mAb combination (**Figures 9A–C**). When comparing rabbits pretreated with c-IgG or anti-Hla/Luk/ClfA mAb combination at the terminal endpoint, significant differences were noted with cardiac troponin I (multiplicity-adjusted *P*<0.01), but not CK-MB or myoglobin (**Figures 9A–C**). The aspartate aminotransferase to alanine aminotransferase (AST/ALT) ratio increased to greater than 5 in 44% (4/9) rabbits pretreated with c-IgG compared to 0% (0/10) for those pretreated with anti-Hla/Luk/ClfA mAb combination (**Figure 9D**). In the four c-IgG-pretreated rabbits with AST/ALT >5, ALT was only mildly elevated (suggestive of minimal liver necrosis) whereas AST was markedly elevated (suggestive of myocardial necrosis) (47), which is consistent also with their markedly elevated levels of cardiac troponin I, CK-MB, and myoglobin (**Figures 9A–C**). Creatinine and blood urea nitrogen levels were mildly elevated in only 22% (2/9) and 10% (1/10) of rabbits pretreated with c-IgG and anti-Hla/Luk/ClfA mAb combination (two-sided Fisher’s exact test, *P* = 0.58;**Figures 9E–F**), suggesting minimal impact of USA300-induced septic shock on kidney function.

## Discussion

Our new mechanically ventilated rabbit model (**Figure 1**) reproduced an extended hyperdynamic state of septic shock that was characterized by elevated cardiac output, increased stroke volume, and reduced systemic vascular resistance, which was then followed by a short-lived and lethal hypodynamic state characterized by rapid decline in mean arterial pressure, increased in central venous pressure, reduced cardiac output, reduced stroke volume, elevated systemic vascular resistance (**Figure 2**), and reduced left-ventricular ejection fraction (**Figure 3**). The hyperdynamic and hypodynamic states of septic shock observed in this rabbit model closely mimicked the hallmark clinical features of human septic shock (30, 48), which may prove to be critical for using this preclinical animal model as a tool to help predict clinical efficacy of novel anti-staphylococcal drug candidates.

Because the development of a hyperdynamic state in patients with septic shock and in preclinical animal models is dependent on adequate fluid resuscitation (30–34), volume repletion in our rabbit model was implemented by infusion of 60-65 mL/kg of a balanced crystalloid solution over the course of 6 h preceding *S. aureus* challenge (**Figure 1A**). Our non-aggressive fluid repletion approach allowed for the development of a hyperdynamic state of septic shock as evidenced by the increased stroke volume observed in all (6/6) rabbits as early as 2 h after *S. aureus* challenge (**Figures 2A–F**), suggesting that adequate volume repletion was administered (49, 50). It should be noted that our goal is to model in rabbits the transition from pre-sepsis to the development of *S. aureus*-induced hyperdynamic septic shock, and, as such, volume repletion in our rabbit model was implemented before infectious challenge to enable the maintenance and increase in stroke volume that occurred shortly after intravenous injection of *S. aureus*. This experimental setup allowed us to then evaluate whether prophylactic administration of anti-Hla/Luk/ClfA mAb combination prevents the development of *S. aureus* hyperdynamic and hypodynamic septic shock in the setting of pre-administration of volume repletion.

We showed here in this new rabbit model of hyperdynamic septic shock that prophylaxis with the anti-Hla/Luk/ClfA mAb combination resulted in a 69% greater survival rate compared to prophylaxis with c-IgG (**Figure 4A**), and that improved survival was associated with its protection against *S. aureus*-induced acute circulatory failure (**Figure 5**). The most severe manifestation of acute circulatory failure, aside from sudden death, is septic shock, which can be difficult to characterize in the critically ill patients because of modifying factors such as antibiotic therapy and supportive care (48). In our mechanically ventilated rabbit model, the hallmark clinical features of septic shock are readily recognized in rabbits pretreated with c-IgG. USA300-induced severe hypotension (**Figure 5A**) was associated with extreme hyperlactatemia (**Figure 8D**), a biomarker of global tissue hypoxia/hypoperfusion (51) that occurred despite adequate arterial oxygenation (**Figure 6B**) from our lung-protective low-tidal volume mechanical ventilation strategy. The latter stages of USA300-induced septic shock in the rabbit model were further characterized by hypocapnia (**Figure 8A**), hyperkalemia (**Figure 8F**), leukopenia (**Figure 7A**), neutropenia (**Figure 7C**), monocytopenia (**Figure 7E**) and lymphopenia (**Figure 7G**), which are common components of human septic shock (52, 53). Prophylaxis with anti-Hla/Luk/ClfA mAb combination, however, halted the progression of acute circulatory failure in 80% (8/10) of rabbits (**Figure 5B**), thereby preventing marked changes to these biomarkers and their grave prognosis. Importantly, the improved survival associated with anti-Hla/Luk/ClfA mAb combination may be due in part to its capacity to reduce growth and survival of USA300 in the heart (**Figure 4B**) and protect against acute myocardial injury (**Figures 9A–C**).

Our study has limitations. First, our sample size calculation was powered to detect as statistically significant a 60% difference in survival (the primary study outcome), requiring a small sample size of only nine animals per experimental group. As such, our study was not powered for statistical analysis of secondary study outcomes comparing changes in blood biomarkers that also requires accounting for multiple comparisons. Second, we did not determine the individual contributions of each mAb for protection against USA300-induced septic shock in the present study. However, it should be noted that these three mAbs when administered alone or in various combinations had demonstrated efficacy in the other preclinical models, including mouse, ferret and rabbit models of pneumonia (54–56), mouse and rabbit models of dermonecrosis (57, 58), mouse models of surgical site infection (16), a diabetic mouse wound infection model (17), and a rabbit model of prosthetic joint infection (18). Third, only the community-associated MRSA USA300 strain was used in the rabbit model of septic shock for efficacy testing because it expresses all the virulence factors targeted the anti-Hla/Luk/ClfA mAb combination. Future studies would characterize the efficacy of this mAb combination against other clinically relevant MRSA strains, including some hospital-associated MRSA strains that do not express PVL. Fourth, while we demonstrated efficacy of anti-Hla/Luk/ClfA mAb combination for pre-exposure prophylaxis, we did not address its efficacy in post-exposure exposure treatment as an adjunctive therapy to the standard of care, which includes antibiotic, fluid resuscitation and vasopressor that are vital to the management of patients with *S. aureus* septic shock. For future post-exposure treatment studies in the rabbit model, aggressive fluid resuscitation may be triggered at a later time points after *S. aureus* challenge using precise thresholds for acute hemodynamic deterioration (e.g., when MAP decreased >20% from pre-infection baseline), which aligns better with the recommendation in the Surviving Sepsis Campaign guidelines that initial fluid resuscitation begins immediately on diagnosis of sepsis and organ dysfunction (48).

In summary, prophylactic administration of the anti-Hla/Luk/ClfA mAb combination protected against acute circulatory failure and death in a rabbit model of USA300-induced hyperdynamic septic shock. Prognostic biomarkers associated with improved survival in this rabbit model may be useful for designing clinical trials to evaluate efficacy of the anti-Hla/Luk/ClfA mAb combination against *S. aureus* severe sepsis and septic shock.

## Data availability statement

The raw data supporting the conclusions of this article will be made available by the authors, without undue reservation.

## Ethics statement

The animal study was approved by University of California San Francisco Institutional Animal Care and Use Committee. The study was conducted in accordance with the local legislation and institutional requirements.

## Author contributions

NN: Data curation, Formal Analysis, Investigation, Methodology, Writing – review & editing. TD: Data curation, Formal Analysis, Methodology, Investigation, Writing – review & editing. KS: Investigation, Writing – review & editing. CT: Investigation, Writing – review & editing, Methodology, Resources. BS: Resources, Writing – review & editing, Conceptualization, Project administration, Supervision. BD: Conceptualization, Project administration, Resources, Supervision, Writing – review & editing, Data curation, Formal Analysis, Funding acquisition, Investigation, Methodology, Visualization, Writing – original draft.
